# Phenotype and genotype of FXIII deficiency in two unrelated probands: identification of a novel *F13A1* large deletion mediated by complex rearrangement

**DOI:** 10.1186/s13023-019-1144-z

**Published:** 2019-07-24

**Authors:** Siyu Ma, Changming Chen, Qian Liang, Xi Wu, Xuefeng Wang, Wenman Wu, Yan Liu, Qiulan Ding

**Affiliations:** 1 State Key Laboratory of Medical Genomics, Shanghai Institute of Hematology, Ruijin Hospital, Shanghai Jiaotong University School of Medicine, 197 Ruijin Second Road, Shanghai, 200025 China; 2 Department of Laboratory Medicine, Ruijin Hospital, Shanghai Jiaotong University School of Medicine, 197 Ruijin Second Road, Shanghai, 200025 China; 3 Faculty of Medical Laboratory Science, Ruijin Hospital, Shanghai Jiaotong University School of Medicine, 197 Ruijin Second Road, Shanghai, 200025 China; 4 Collaborative Innovation Center of Hematology, Shanghai Jiaotong University School of Medicine, Shanghai, China; 5 Department of Burns and Plastic Surgery, Ruijin Hospital Affiliated to Shanghai JiaoTong University School of Medicine, Shanghai, China

**Keywords:** FXIII deficiency, *F13A1*, Large deletion, Genomic rearrangement

## Abstract

**Background:**

Inherited Factor XIII deficiency (FXIIID) is one of the most severe and under-diagnosed rare bleeding disorders. Only 5 large deletions involving one or more exons in *F13A1* have been reported, and lacking of multiplex ligation-dependent probe amplification (MLPA) assay might underestimate the copy number variations (CNVs) in *F13A1* and *F13B*. We had characterized the clinical presentation of two unrelated severe FXIIID probands and explored the pathogenic mechanisms.

**Results:**

Both probands experienced several episodes of fatal bleeding and delayed wound healings prior to diagnosis. FXIII activity was measured by the ammonia release assay, and FXIII-A and FXIII-B antigens were determined by ELISA. All the exons including exon-intron boundaries and promoter regions of *F13A1* and *F13B* were amplified and directly sequenced. Copy number variations (CNVs) of *F13A1* and *F13B* were detected by the CNVplex® method. Breakpoints of the *F13A1* large deletion were identified by quantitative primer walking combined long-range PCR (LR-PCR) strategies. Proband 1 was found to have compound heterozygous mutations of a novel small deletion (c.1147del) and a missense mutation p.Arg383Ser. Proband 2 was compound heterozygous for a novel large deletion (g.[77815_112815del;112837_116628del]) and a missense mutation p.Arg716Gly in *F13A1*. Bioinformatics analysis of the large deletion breakpoints predicted that two fork stalling and template switching and/or microhomology-mediated break-induced replication (FoSTeS/MMBIR) events with two homologies of TCT and C might be responsible for the complex rearrangement. Prophylactic replacement therapy was immediately administered for the two probands upon establishment of the diagnosis.

**Conclusions:**

We detected two type I FXIIID pedigrees and adopted CNVplex® method to detect CNVs of *F13A1* and *F13B* for the first time. A large heterozygous deletion of g.[77815_112815del;112837_116628del] in *F13A1*, mediated by two FoSTeS/MMBIR events, was identified.

**Electronic supplementary material:**

The online version of this article (10.1186/s13023-019-1144-z) contains supplementary material, which is available to authorized users.

## Background

Factor XIII (FXIII), also known as fibrin stabilizing factor, circulates as a non-covalent heterotetramer consisting of two catalytic A subunits (FXIII-A_2_) and two carrier B subunits (FXIII-B_2_) in plasma, whereas intracellular FXIII exists in the form of FXIII-A_2_ dimers [1, 2]. FXIII zymogen is activated into its enzyme form FXIIIa, by thrombin in the presence of Ca^2+^.The cleavage of the activation peptide results in A and B subunits dissociation, which is accelerated by the presence of fibrin [3]. The main function of FXIIIa is to covalently cross link fibrin fibers to stabilize the fibrin clot. In addition, it cross-links fibrinolytic inhibitors such as α2 plasmin inhibitor into the fibrin to modify the clot structure, conferring resistance to premature fibrinolysis. Recent studies have revealed that FXIII performs multifunction not only in the coagulation reactions, but also in several other vital biological processes including angiogenesis, wound healing, maintenance of pregnancy and vascular permeability [4].

Hereditary FXIII deficiency (FXIIID) is a rare bleeding disorder with a prevalence of one per two million individuals. FXIIID is classified into type I and type II FXIII-A deficiency (FXIIID-A) and FXIII-B deficiency (FXIIID-B). Type I FXIIID-A is a quantitative deficiency of FXIII-A, while type II is a functional defect of FXIII-A with almost normal concentration [5]. The typical clinical manifestations of severe FXIIID are neonatal umbilical cord bleeding, intracranial hemorrhage (ICH) and recurrent early miscarriages [6, 7]. If the diagnosis of severe FXIIID is confirmed, prophylactic replacement therapy is mandatory to avoid life-threatening bleeding events. According to the Human Gene Mutation Database Professional (HGMD Professional), a total of 203 mutations have been reported worldwide so far in patients with FXIIID, of which more than 90% of mutations were identified in *F13A1* while only 20 mutations in *F13B.* The spectrum of mutation is constituted with primarily missense/nonsense mutations in *F13A1*, and a few small deletions/insertions and splice site mutations. Only 5 large deletions and one large duplication involving one or more exons in *F13A1* have been reported [8–13]. Because multiplex ligation-dependent probe amplification (MLPA) or other CNV detection methods were not available for screening large deletions or duplications in *F13A1* and *F13B*, the number of these variants might be underestimated. Recently, a next generation sequencing (NGS) panel involving *F13A1* and *F13B* was established, which can screen CNVs and other mutations [14].

Large genomic deletions might occur during the repair of double strand breaks (DSBs) in DNA explained by some proposed mechanisms including non-allelic homologous recombination (NAHR), non-homologous end-joining (NHEJ) or microhomology-mediated end-joining (MMEJ), and fork stalling and template switching and/or microhomology-mediated break-induced replication (FoSTeS/MMBIR) [15–18]. Only one large deletion involving the entire exon 12 of *F13A1* was characterized the breakpoints, and MMEJ mediated genomic rearrangement was proposed [12].

In this study, the clinical and laboratory phenotypes of two unrelated severe FXIIID patients were presented. In addition, comprehensive genetic analysis of *F13A1* and *F13B* was performed using directly sequencing and CNVplex® detection. A novel heterozygous large deletion from exon 7 through exon 8 and the related breakpoints in *F13A1* were identified and a complex rearrangement mechanism was proposed.

## Materials and methods

### Clinical presentation

**Fig. 1:**
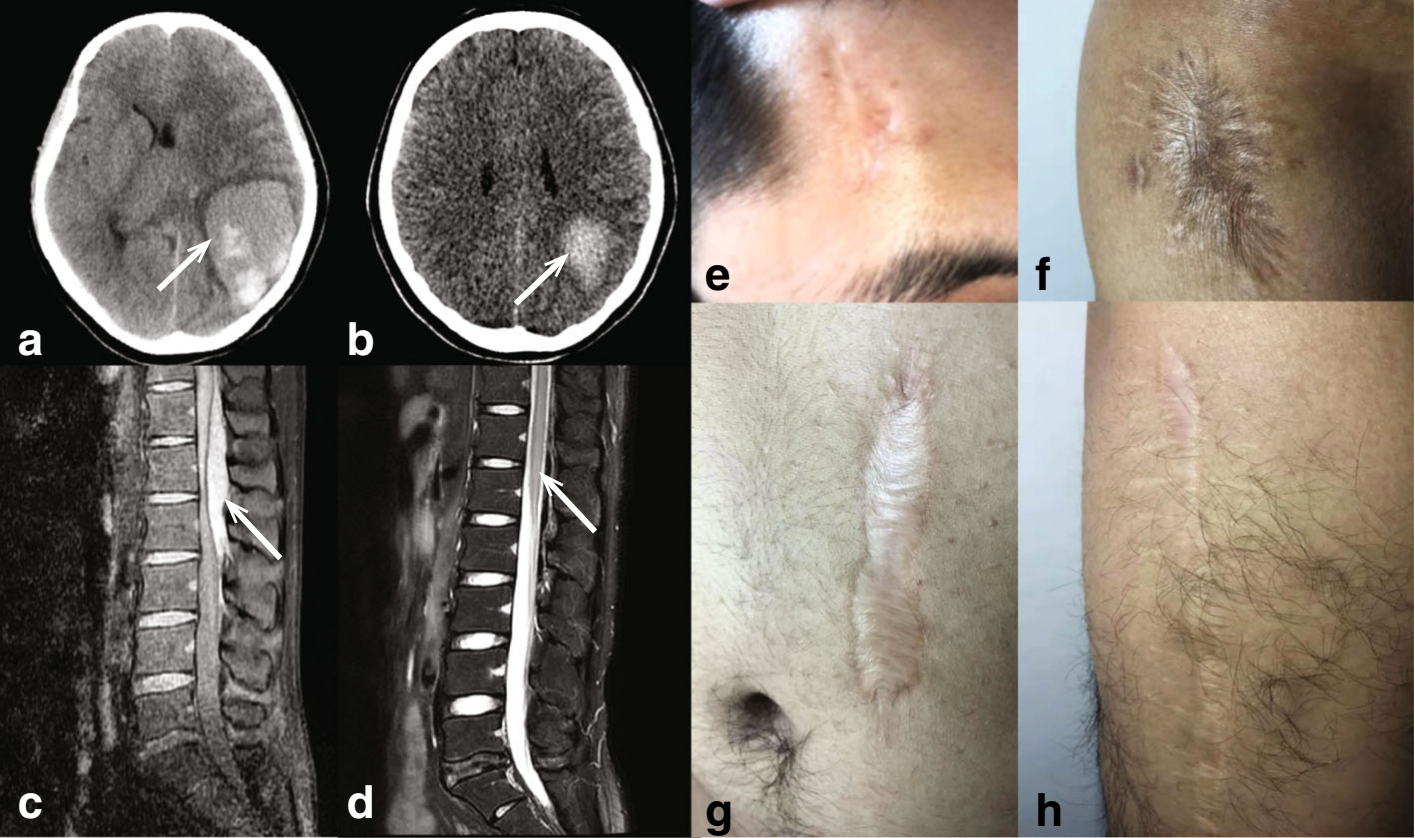
Fetal bleedings and delayed wound healings in probands 1 and 2. **a** and **b** Cerebral hemorrhage of the left top occipital lobe at the beginning and 17 days later, respectively shown by computed tomography (CT) in proband 1, indicated by the arrows; **c** and **d** Spinal cord hematoma located at T12-L2 at the time of diagnosis and 4 days later, respectively, revealed by magnetic resonance imaging (MRI) in proband 2, indicated by the arrows; **e**-**h** Four wounds resulting from delayed wound healing in proband 2. **e** Forehead; **f** Right hip; **g** Left abdomen; **h** Right thigh

Proband 1, a 17-year-old girl, was born by vaginal delivery with umbilical cord hemorrhage, and subsequently, a tendency of ecchymosis presented. She had suffered from muscle hematomas after intramuscular vaccine injections during her childhood. She experienced a spontaneous ICH when she was 4 years old after a collision (no detail clinical information provided), and suffered from left ovarian corpus luteum rupture twice at her 12 and 14 years old, however, she had a normal menstrual cycle and did not complaint about menorrhagia. She also had hemarthrosis in the right hip joint 1 day later after trauma, which required drainage to relieve the swelling and pain. She was diagnosed with low Von Willebrand factor (VWF) level when she was 15 years old, subsequently, her recurrent spontaneous cerebral hemorrhage of the left top occipital lobe was effectively treated with cryoprecipitate (Cryo) infusion at a local hospital and she was completely recovered with no vision impaired (Fig. 1a, and b). The patient was referred to our center for hematology consulting, and her diagnosis of severe FXIIID was established. She was placed on prophylaxis treatment with 600 ml frozen plasma (FP) per month and experienced mild allergic reactions with urticaria occasionally (both plasma-derived FXIII (pd-FXIII) and recombinant FXIII (rFXIII) are unavailable in China). No severe bleeding episodes have happened since then. She is an offspring of non-consanguineous parents with no family history.

Proband 2, a 25-year-old male, has experienced multiple severe bleedings that needed surgical interventions since a young age. A forehead hematoma following a minor injury was recorded at age 5. He suffered from splenic rupture after abdominal trauma and emergent splenectomy was performed to control the severe retroperitoneal hemorrhage at age 8. He suffered from trauma-related episodes of the right thigh muscle hematoma and hemarthrosis in his right hip joint, and both were treated by drainage. The prolonged wound healing, characterized by one-month-long blood oozing from injured lesions, was encountered on all episodes of trauma detailed above (forehead, abdomen, right thigh and right hip) and almost no transfusion was administrated during these wound healing periods. The Vancouver Scar Scale (VSS) was used to assess the severity of each of the four wound scars, and the scores ranged from 2 to 3, and the proband reported no signs of pain and itch of scars (Fig. 1e-h). He had spontaneous recurrent hematuria without any urinary system disease and recurrent trauma-related hemarthrosis in various joints with no joint deformities observed so far. Later, he had a dull low back pain lasting 1 day and suddenly became to sharp pain, and he was immediately admitted into the hospital. Physical examination showed tenderness on the thoracic-lumbar area, and no neurological deficit was found. MRI scan revealed a 7.5 cm × 1.0 cm spinal cord hematoma at T12-L2 levels (Fig. 1c, and d). Considering his bleeding history, he was treated with FP, his symptoms improved gradually and he was discharged from the hospital 1 week later with complete recovery. He was diagnosed with severe FXIIID in our center after that episode. FP prophylaxis treatment (600 ml per month) was taken. Slight itching all over the body and occasional urticarial rash were presented but quickly disappeared approximately 4 h later after transfusion, and no severe bleeding episodes happened so far. He was born to non-consanguineous parents with no family history.

### FXIII and VWF assays

Peripheral blood was collected from the affected individuals and related family members. Platelet-poor plasma (PPP) and platelet-rich plasma (PRP) were prepared as previously described [19]. Coagulation screen tests including activated partial thromboplastin time (APTT), prothrombin time (PT), thrombin time (TT) and fibrinogen (FIB) in PPP were performed on a CA7000 automatic coagulometer (Sysmex, Kobe, Japan) as routine protocols. Platelet aggregation in PRP with ristocetin at final concentrations of 0.5 mg/ml and 1.2 mg/ml or adenosine diphosphate (ADP) at 2umol/L was performed on a Chrono-log aggregometer model 560 (Chrono-log Corporation, Harvertown, USA). VWF antigen (VWF:Ag), VWF activity (VWF:Ac) and FVIII activity (FVIII:C) were also assayed as preciously described [19]. ABO-blood-type was detected as routine protocols.

The diagnosis and classification of FXIIID were established following the recommendations of the Standardization and Scientific Committee of the International Society on Thrombosis and Haemostasis (ISTH SSC) [5]. The quantitative functional FXIII activity (FXIII:Act) assay was determined with a spectrophotometric ammonia release assay using a BIOPHEN Factor XIII kit (HYPHEN BioMed, Neuville-sur-Oise, France). FXIII-A antigen (FXIII-A:Ag) was measured using a sandwich ELISA method with a sheep anti-FXIII-A polyclonal antibody (Enzyme Research laboratories, South Bend, USA) as a capture antibody and horseradish peroxidase (HRP) labeled antibody as a detection antibody, and FXIII-B antigen (FXIII-B:Ag) was measured with an anti-FXIII-B polyclonal antibody ELISA detection kit (Cusabio Biotech Co. Ltd., Wuhan, China). The mixing study was performed on samples with positive findings to differentiate between congenital FXIIID and the presence of FXIII inhibitor.

### Genetic analysis of *F13A1* and *F13B*

DNA was extracted from peripheral blood leukocytes using the QIAamp DNA extraction kit (Qiagen, Hilden, Germany). All the exons along with flanking sequences, and 5′ and 3′ untranslated regions (5′ UTR and 3′ UTR) of *F13A1* and *F13B* were amplified by PCR and sequenced directly.

CNVs of *F13A1* and *F13B* were analyzed by the CNVplex® technique (Genesky Biotechnologies, Shanghai, China), a high-throughput multiplex CNV detection method [19]. Using synthetic oligonucleotides, 63 target-specific probe pairs were selected including 39 pairs in *F13A1* with five pairs on the 5′ UTR, two pairs on each of 15 exons and four pairs on the 3′ UTR, and 24 pairs covering all 12 exons with two pairs on each of the exons in *F13B* (Additional file 1: Table S1). In addition, 32 reference probes located in different sub-chromosomal loci presented no reported copy number polymorphisms were selected (data not shown). The target sequences of each probe were listed in Additional file 1: Table S1. Amplification products were loaded on an ABI 3130xl Genetic Analyser (Applied Biosystems, Foster City, USA) for capillary electrophoresis analysis. Raw data were analyzed by GeneMapper 4.0 (Applied Biosystems) as previously described [19].

The *F13A1* and *F13B* mutations were designated based on the cDNA sequence (Genbank NM_000129.3 and NM_001994.2) and referred to the recommendation of Human Genome Variation Society with nucleotide + 1 as the A of the ATG translation initiation codon [20]. Only the mutation-corresponding sequence was detected in related family members.

### The breakpoint analysis of the large deletion of *F13A1*

**Fig. 2:**
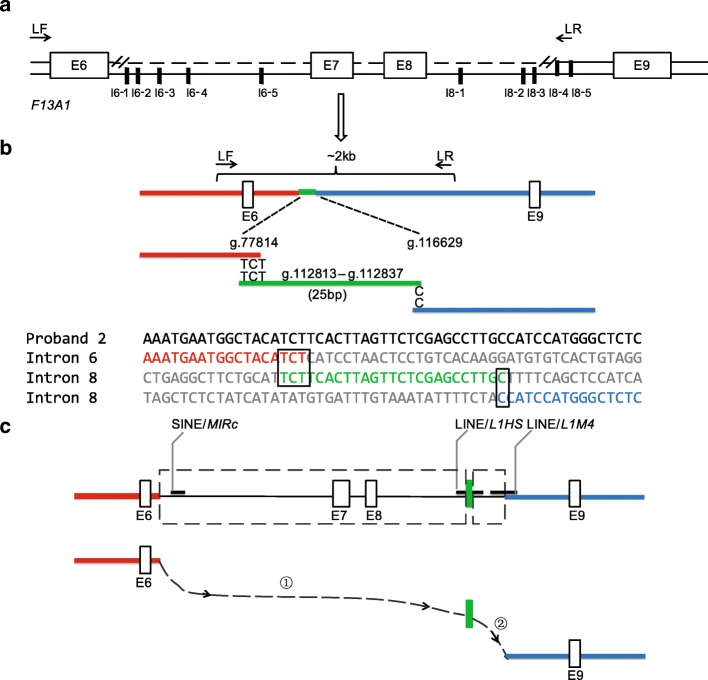
Breakpoint detection and rearrangement mechanism of the large deletion of *F13A1* in proband 2. **a** Breakpoint detection of the large deletion of *F13A1* by quantitative primer walking combined with LR-PCR strategies; **b** Identification of the breakpoints by sequencing of the LR-PCR product; **c** The complex rearrangement mediated by two FosTes/MMBIR events with two micro-homologies of TCT and C, respectively. Ten fragments used in primer walking detection were indicated by black blocks, and deletion by two dotted line rectangular frames; LF and LR were the forward and reverse primers for the LR-PCR amplification. The two micro-homologies of TCT and C were indicated by two rectangular frames, respectively. SINE/*MIRc*, LINE/*L1M4* and LINE/*L1HS* were indicated by black bars

As the CNVs analysis of the *F13A1* identified the heterozygous deletion from exon 7 through exon 8 in proband 2, quantitative primer-walking combined long-range PCR (LR-PCR) strategies were used to localize the breakpoints between intron 6 and intron 8 (Fig. 2a). Firstly, the primer walking strategy was applied to narrow down the regions of breakpoints with 5 fragments (I6–1 to I6–5) located in intron 6 and 5 fragments (I8–1 to I8–5) in intron 8 using quantitative PCR (qPCR) (primers sequences shown in Additional file 1: Table S2). RNA polymerase II subunit A (*POLR2A*), T-box 15 (*TBX15*), and ribonuclease P/MRP subunit p14 (*RPP14*) were used as reference genes of R1, R2 and R3, respectively (Additional file 1: Table S2). A DNA sample from one normal individual was used as a normal control. As shown in Fig. 2b, specific LR-PCR flanking the predicted two breakpoints was subsequently applied to precisely identify the breakpoints with the Phusion High-Fidelity DNA Polymerase (Thermo Scientific, Massachusetts, USA), and DNA of a normal individual was used as a subsequently negative control (primers sequences shown in Additional file 1: Table S2). The PCR products were analyzed by electrophoresis and sequenced.

### The effect of the large deletion of *F13A1* on transcription

To analyze the effect of the chimeric intron, consisting of part of intron 6 linked to two separate parts of intron 8 generated by the large deletion (Fig. 2c), on *F13A1* mRNA transcription, total mRNA was extracted from peripheral blood leukocytes of proband 2 and one normal control, and the reverse transcription PCR (RT-PCR) was performed as previously described [21]. The mRNA transcript from exon 4 through exon 11 of *F13A1* was amplified using forward primer covering the boundary of exons 3 and 4, and reverse primer covering the boundary of exons 11 and 12 to exclude the possible interference of DNA (Additional file 1: Table S4). PCR products were analyzed by electrophoresis on the 1% agarose gel and directly sequenced.

Proband 2 was a heterozygote of the SNP (c.1694C > T; rs5982), which is located in exon 12 of *F13A1* and with SNP-C linked to the large deletion allele revealed by segregation analysis. Allelic imbalance was detected using a primer extension-based method [22]. A short *F13A1* cDNA fragment covering the SNP was amplified with a pair of primers (Additional file 1: Table S4) using DNA and cDNA as templates from the proband 2 and a normal control with the same SNP genotype. Then the amplicons were purified and amplified with an extension primer, designed to anneal one base upstream of the SNP site, using the SNaPshot Multiplex kit (Applied Biosystems). The SNP was detected by enzymatic elongation of the primer by a SNP-complementary, fluorescently labeled dideoxy-nucleoside triphosphate (ddNTP) on an ABI 3730xl Genetic Analyser (Applied Biosystems). The data were analyzed using the GeneMapper 4.1 software program (Applied Biosystems).

### Bioinformatics analysis

In the search of homologous sequences, alignment of sequences up to 10 kb from the breakpoints was filtered by the basic local alignment search tool BLAST of the NCBI and EMBOSS Sequence Alignment [23, 24]. The 1000 bp reference sequence flanking both proximal and distal breakpoints was searched by Repeat Masker for repetitive elements [25], which were associated with rearrangement mechanisms [26], such as long interspersed nuclear elements (LINEs), short interspersed nuclear elements (SINEs), long terminal repeats (LTRs) and DNA transposons.

Sequences located within 50 bp from the breakpoints were analyzed for the presence of DNA motifs (and their complements) that are known to be associated with genomic deletions. Non-B DNA conformations, which can stimulate the formation of DSBs and generate genomic rearrangement [27], were screened using REPEATAROUND and QGRS MAPPER [28, 29]. REPEATAROUND was used to identify direct repeats, mirror repeats and inverted repeats. QGRS MAPPER screens G-rich sequence, which was likely to induce tetraplex formation of one strand of DNA with another unpaired single strand. FUZZNUC was applied to screen topoisomerase sites and translin-binding sites, which were related to copy number variations [30].

## Results

### FXIII and VWF assays

**Fig. 3:**
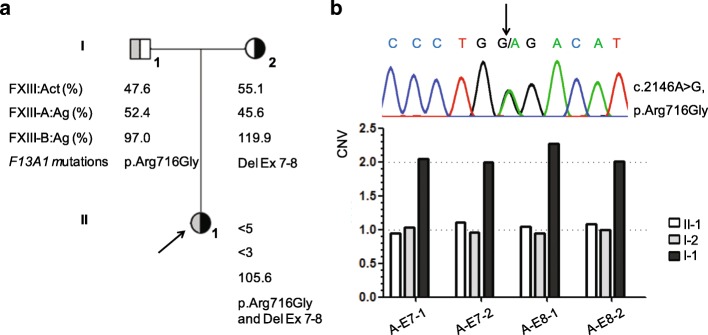
Phenotype and genotype results of pedigree 2 with severe type 1 FXIIIA deficiency. **a** Segregation analysis of pedigree 2; **b** Compound heterozygous mutations (p.Arg716Gly and Del Ex 7–8) of *F13A1* in proband 2. Del Ex 7–8, the *F13A1* large deletion from exon 7 through exon 8; A-E7–1 and A-E7–2, two fragments located in exon 7 of *F13A1*; A-E8–1 and A-E8–2, two fragments located in exon 8 of *F13A1*; CNV, copy number variation

Routine coagulation tests and the platelet aggregation assay revealed no abnormalities in both patients (data not shown). Both probands had significantly low levels of FXIII:Act and FXIII-A:Ag (FXIII:Act < 5% and FXIII-A:Ag < 3% of normal levels), whereas the levels of FXIII-B:Ag were normal (115.9% in proband 1 and 105.6% in proband 2). The mixing assay was negative in both probands. Proband 1 and her mother were with ABO blood type O and low levels of VWF:Ag and VWF:A in parallel. Therefore, both probands 1 and 2 were diagnosed as severe type I FXIIID-A. In addition, proband 1 was combined with low level of VWF. The data of FXIII levels in the two pedigrees were listed in Table 1 and Fig. 3.

**Table 1:** Phenotype and genotype results of pedigree 1 with severe type 1 FXIIIA deficiency

	Sex/year	ABO blood type	VWF:Ag (%)	VWF:A (%)	FVIII:C (%)	FXIII:Act (%)	FXIII-A: Ag (%)	FXIII-B: Ag (%)	*F13A1* mutations
Proband 1	F/15	O	51.4	58.5	85.8	< 5	< 3	115.9	c.1149G > T, p.Arg383Ser and c.1147del, p.Arg383GlyfsTer82
Mother	F/40	O	53.8	52.5	61.4	52.9	40.7	98.7	c.1149G > T, p.Arg383Ser
Father	M/43	O	134.5	123.1	114.3	65.1	57.6	103.1	c.1147del, p.Arg383GlyfsTer82

*M* Male, *F* Female

### Genetic analysis of *F13A1* and *F13B*

Compound heterozygous mutations of a novel small deletion of c.1147del causing frameshift followed by 81 altered amino acids and termination codon, and a previously reported missense mutation of p.Arg383Ser in *F13A1* were identified in proband 1, originated from her father and mother, respectively (Table 1). Only a previously reported heterozygous missense mutation of p.Arg716Gly was found in proband 2 by directly sequencing. However, the CNVplex® assay detected a novel large heterozygous deletion of exon 7 through exon 8 in *F13A1*. Segregation analysis showed that the missense mutation and large deletion were inherited from his father and mother, respectively (Fig. 3). No causative mutation was identified in *F13B* in both probands. In addition, two heterozygous single nucleotide polymorphisms (SNPs) of Pro565Leu in *F13A1* and Arg115His in *F13B* were found in both probands.

### The breakpoints analysis of the large deletion

As shown in Fig. 3a and Additional file 1: Table S3, the quantitative primer walking strategy revealed that each of 5 fragments (I6–1 to I6–5) located in intron 6 and 3 fragments (I8–1 to I8–3) in intron 8 presented one copy and each of 2 fragments (I8–4 to I8–5) in intron 8 presented two copies, suggesting the two breakpoints localized between exon 6 to I6–1 and between I8–3 to I8–4, respectively. Specific LR-PCR was used to amplify the flanking areas of the two breakpoints and two precise breakpoints were identified by directly sequencing: one located at g.77814 in intron 6 and the other at g.116629 in intron 8. As shown in Fig. 3b, there were nucleotides, matching to g.112813_g.112837 in intron 8, exactly connected to two breakpoints with 3 bp of TCT proximal and 1 bp of C distal microhomologies. Therefore, the large heterozygous deletion of *F13A1* designated as g.[77815_112815del;112837_116628del] was identified in proband 2.

### The effect of the large deletion of *F13A1* on transcription

As shown in Additional file 1: Figure S1, no abnormal *F13A1* mRNA transcript but only the normal transcript was observed by electrophoresis of the RT-PCR product, and the normal transcript was confirmed by directly sequencing in proband 2 (data not shown), indicating that the abnormal mRNA chain may be degraded. Allelic imbalance detection showed that the peak height of SNP-C was significantly decreased, whereas there was no obvious difference in the SNP-T in the mRNA transcript compared to that in DNA, further indicating the disappearance of the allele with the large deletion in proband 2 (Additional file 1: Figure S1). Altogether, the mRNA analysis indicated the presence of nonsense-mediated mRNA decay (NMD) caused by the large deletion of *F13A1*.

### Bioinformatics analysis

Sequence homologies at or near both breakpoint regions were not identified by the BLAST screen. RepeatMasker analysis showed three retrotransposons: one Mammalian-wide Interspersed Repeat-variant c (SINE/MIR *MIRc*) 62 bp downstream of the proximal breakpoint, one L1 Mammalian group 4 (LINE/L1 *L1M4*) over the distal breakpoint, and one L1 *Homo sapiens* (LINE/L1 *L1HS*) covering the 25 bp insertion (Fig. 2c). The sequence similarity between LINE/L1 *L1M4* and LINE/L1 *L1HS* was only 45.2%. Therefore, the large deletion from exons 7 through 8 was not mediated by NAHR with homologies or repetitive elements. As shown in Fig. 2b, there was a 25 bp sequence alignment to intron 8 of *F13A1* inserted between the two breakpoints with one 3-bp microhomology (TCT) at the proximal breakpoint in intron 6 and another one 1-bp microhomology (C) at the distal breakpoint in intron 8. Bioinformatics analysis predicted that the complex rearrangement might be generated by two FoSTeS/MMBIR events with two microhomologies of TCT and C (Fig. 2c).

As shown in Additional file 1: Figure S2, three direct repeats (dr) were screened with two located on each side of the proximal and distal breakpoints, respectively, and the other located downstream of the 25 bp insertion. Two inverted repeats (ir) on both sides of the two breakpoints were identified.

## Discussion

FXIIID is one of the most under-diagnosed rare bleeding disorders, especially in developing countries, where only a few cases with severe FXIIID have been reported so far [31–33]. In addition to lacking awareness of this rare disease, the missed or delayed diagnosis of FXIIID is partly attributed to inaccessibility of specific quantitative FXIII:Act and FXIII:Ag assays recommended by ISTH SSC to confirm the FXIIID diagnosis. Although having experienced several typical bleeding episodes and consulted doctors in local hospitals, the two unrelated probands investigated in current study did not have the diagnosis of severe FXIIID established until adult age in our haemophilia center.

FXIIID is one of the most severe bleeding disorders and causes highest incidence of ICH (~ 30%) among all congenital bleeding disorders. Though ICH often occurs after trauma in children, it frequently presents spontaneously in adults causing as high as 15% mortality [34]. Both probands described here experienced severe bleedings including ICH and ruptures of spleen. Notably, proband 2 suffered rare intramedullary spinal cord hematoma, which had been found as complications to haemophilia and congenital factor XI deficiency [35, 36]. To the best of our knowledge, this is the first report of spinal cord hematoma in FXIIID patients. FXIII has very long half-life (11~14 days) and very low level of FXIII (> 3–10%) is already sufficient for bleeding control. Therefore, if the diagnosis of severe FXIIID is confirmed, prophylactic replacement therapy is mandatory. The two probands had not experienced major bleeding episodes since the prophylactic therapy was administered. The improved awareness of health care providers and accessibility of laboratory assays should be essential to the early diagnosis and management of severe FXIIID and to reduce its morbidity and mortality in developing countries.

FXIIID associated with impaired wound healing was first reported in 1960 in a seven-year old Swiss FXIIID boy with subsequently extensive, irregular, and retracted scars after surgical intervention [37]. Defective wound healing mainly mentioned as prolonged wound healing has been reported in 14–29% of FXIIID patients [6, 7]. Interestingly, proband 2 presented clearly delayed wound healings recurrently in each of four wounds with blood oozing from the injured lesions. Few plasma or Cryo products were administrated after trauma or during the period of surgical interventions. However, the scores of each of the four wound scars ranged from 2 to 3 evaluated by VSS, and he did not complain of pain and itching over these scars. FXIII belongs to one of the transglutaminase family members, and like other homologous tissue transglutaminase, it plays significant roles in tissue repair and wound healing [38–41]. Clinical experience and animal experiments appear to indicate that, in order to obtain satisfactory healing of surgical wounds and avoid potential complications of wound healing, sufficient levels of hemostasis are necessary for 2–3 weeks after surgery [42]. Therefore, we predicted that the impairment in clot stabilization, which is the unique and irreplaceable function of FXIII, should be mainly responsible for the prolonged wound healing in FXIIID. The severity of scars evaluated by scar assessment scales in more FXIIID patients may provide novel insights to reveal the role of FXIII in wound healing.

FXIIID causing mutations in *F13A1* always lead to complete absence of the protein associated with severe FXIIID in homozygous or compound heterozygous patients [43]. Here, in addition to two previously reported recurrent missense mutations (p.Arg383Ser and p.Arg716Gly), both of which had been confirmed to be null mutations [44, 45], two novel heterozygous deletions (c.1147del and g.[77815_112815del;112837_116628del]) of *F13A1* were identified in the two probands in current study. Nonsense-mediated mRNA decay (NMD) was reported in some frame shift deletion/insertion or splicing site mutations of *F13A1* as determined by quantitative RT-PCR analysis [10, 46]. The large heterozygous deletion of g.[77815_112815del;112837_116628del] of *F13A1* produced a new chimeric intron, which may generate an alternative splicing pattern. However, the mRNA analysis revealed no abnormal transcript, and allelic imbalance detection further confirmed the disappearance of the allele with the large deletion in proband 2, indicating that the large deletion was the causative mutation mediated by NMD.

Remarkably, only five large deletions have ever been reported in *F13A1*. Four large homozygous deletions of exons 3, 5, 12 and 15 in four unrelated patients were found by lack of amplification around this region at the DNA level. The remaining huge heterozygous deletion encompassing exons 4 through 11 was found by detecting an open reading frame-maintaining mRNA transcript by RT-PCR [8]. In the current study, we adopted CNVplex® method to detect CNVs of *F13A1* and *F13B* for the first time and identified a large heterozygous deletion in *F13A1* in proband 2.

Molecular mechanisms responsible for nonrecurrent rearrangements could be explained by homology-dependent NAHR and nonhomologous repair mechanisms such as classical NHEJ or NHEJ mediated by microholomogy (alt-NHEJ or MMEJ). They could also come into being through replication-based mechanisms such as FoSTeS, which was originally proposed to explain multiple duplications found in Pelizaeus–Merzbacher disease patients, and was subsequently generalized as MMBIR [17, 18]. The FoSTeS/MMBIR have now been increasingly recognized as plausible mechanisms prevailing in rare formation of pathogenic CNVs [47]. It utilizes micro-homologies to switch the template upon replication fork stalling. Replication continues based on a wrong template until the original fork is restored. The template being switched to is usually in physical proximity but not necessarily in close linear proximity to the original replication fork. Such template switching may occur several times before the replication process gets back to its original template, resulting in complex rearrangements.

The large deletion from exons 7 through 8 identified in the current study is unlikely to be mediated by NAHR with homologies or repetitive elements. As shown in Fig. 2c, the large deletion could be formed by two FoSTeS/MMBIR events with two consecutive steps of forward slippage: the first, a longer-linear-distance template switch mediated by misalignment at 3 bp microhomology of TCT; the second, a shorter-distance template switch mediated by misalignment at 1 bp microhomology of C, resulting in a 35 kb and a 3.8 kb deletions, respectively occurring in the complex rearrangement process.

It has been recognized that the CNVs are not randomly distributed, but affected by multiple genomic features. The SINE/*MIRc*, LINE/*LIM4* and LINE/*L1HS* were identified near the breakpoints of the large deletion and these repetitive elements could be attributed to gene rearrangement by stimulating DNA breakage in these areas [15, 26]. In addition, 3 direct repeats and 2 inverted repeats were identified around the breakpoint regions of the large deletion. Non-B DNA structure such as slipped structures and hairpin/cruciforms could be formed by these sequences, which may increase genetic instability and stimulate the formation of DSBs, an obligate step in the generation of gross rearrangements [15, 27]. However, the mechanism of gene rearrangement in *F13A1* mediated or stimulated by gene structure needs to be revealed by more CNVs detection and breakpoint identification in FXIIID patients.

## Conclusion

The diagnosis of severe FXIIID was made by comprehensive phenotype and genotype detections in two unrelated probands, who were immediately treated with prophylactic replacement therapy. The large heterozygous deletion of *F13A1* and the related breakpoints were identified. The bioinformatics analysis of the breakpoints predicted that the complex rearrangement was mediated by two consecutive FoSTeS/MMBIR events. The CNVplex® method for *F13A1* and *F13B* was established for the first time, which would be valuable for CNVs detection in FXIIID, especially in heterozygous mutations.

## Additional file

Additional file 1: **Table S1.** The target sequences for CNV detection of *F13A1* and *F13B* in probands 1 and 2. **Table S2.** Primer sequences for quantitative primer walking detection and LR-PCR amplification. **Table S3.** Quantitative PCR results of 10 fragments in intron 6 and intron 8 of *F13A1* for the primer walking detection in proband 2. **Table S4.** Primer sequences for mRNA analysis of the large deletion of *F13A1*. **Figure S1.** Effect of the *F13A1* large deletion on transcription analyzed by RT-PCR in proband 2. **Figure S2.** Instability analysis of the DNA sequences around breakpoints and insertion in proband 2. (DOCX 4664 kb)

## Data Availability

The datasets supporting the conclusions of this article are included within the article (and its additional files).

## References

[1] Takahashi N, Takahashi Y, Putnam FW (1986). Primary structure of blood coagulation factor XIIIa (fibrinoligase, transglutaminase) from human placenta. Proc Natl Acad Sci U S A.

[2] Carrell NA, Erickson HP, McDonagh J (1989). Electron microscopy and hydrodynamic properties of factor XIII subunits. J Biol Chem.

[3] Lorand L (1986). Activation of blood coagulation factor XIII. Ann N Y Acad Sci.

[4] Muszbek L, Bereczky Z, Bagoly Z, Komaromi I, Katona E (2011). Factor XIII: a coagulation factor with multiple plasmatic and cellular functions. Physiol Rev.

[5] Kohler HP, Ichinose A, Seitz R, Ariens RA, Muszbek L, Factor X (2011). Diagnosis and classification of factor XIII deficiencies. J Thromb Haemost.

[6] Ivaskevicius V, Seitz R, Kohler HP, Schroeder V, Muszbek L, Ariens RA (2007). International registry on factor XIII deficiency: a basis formed mostly on European data. Thromb Haemost.

[7] Viswabandya A, Baidya S, Nair SC, Abraham A, George B, Mathews V (2012). Correlating clinical manifestations with factor levels in rare bleeding disorders: a report from Southern India. Haemophilia.

[8] Anwar R, Miloszewski KJ, Markham AF (1998). Identification of a large deletion, spanning exons 4 to 11 of the human factor XIIIA gene, in a factor XIII-deficient family. Blood.

[9] Ivaskevicius V, Windyga J, Baran B, Schroeder V, Junen J, Bykowska K (2007). Phenotype-genotype correlation in eight polish patients with inherited factor XIII deficiency: identification of three novel mutations. Haemophilia.

[10] Otaki M, Inaba H, Shinozawa K, Fujita S, Amano K, Fukutake K (2008). Characterization of a large deletion that leads to congenital factor XIII deficiency. Rinsho Byori.

[11] Shanbhag S, Ghosh K, Shetty S (2016). Genetic basis of severe factor XIII deficiency in a large cohort of Indian patients: identification of fourteen novel mutations. Blood Cells Mol Dis.

[12] Thomas A, Ivaskevicius V, Zawadzki C, Goudemand J, Biswas A, Oldenburg J (2016). Characterization of a novel large deletion caused by double-stranded breaks in 6-bp microhomologous sequences of intron 11 and 12 of the F13A1 gene. Hum Genome Var.

[13] Inaba H, Shinozawa K, Amano K, Fukutake K (2010). Identification and characterization of li-mediated large tandem duplication, spanning exon 4 to 10 of the F13A1, in a patient with congenital factor XIII deficiency. Blood.

[14] Bastida JM, Del Rey M, Lozano ML, Sarasquete ME, Benito R, Fontecha ME (2016). Design and application of a 23-gene panel by next-generation sequencing for inherited coagulation bleeding disorders. Haemophilia.

[15] Hastings PJ, Lupski JR, Rosenberg SM, Ira G (2009). Mechanisms of change in gene copy number. Nat Rev Genet.

[16] Fujita J, Miyawaki Y, Suzuki A, Maki A, Okuyama E, Murata M (2012). A possible mechanism for Inv22-related F8 large deletions in severe hemophilia a patients with high responding factor VIII inhibitors. J Thromb Haemost.

[17] Lee JA, Carvalho CM, Lupski JR (2007). A DNA replication mechanism for generating nonrecurrent rearrangements associated with genomic disorders. Cell.

[18] Hastings PJ, Ira G, Lupski JR (2009). A microhomology-mediated break-induced replication model for the origin of human copy number variation. PLoS Genet.

[19] Liang Q, Qin H, Ding Q, Xie X, Wu R, Wang H (2017). Molecular and clinical profile of VWD in a large cohort of Chinese population: application of next generation sequencing and CNVplex((R)) technique. Thromb Haemost.

[20] Human Genome Variation Society. http://www.hgvs.org/. Accessed 1 Mar 2019.

[21] Chen C, Xie X, Wu X, Lu Y, Wang X, Wu W (2017). Complex recombination with deletion in the F8 and duplication in the TMLHE mediated by int22h copies during early embryogenesis. Thromb Haemost.

[22] Caux-Moncoutier V, Pages-Berhouet S, Michaux D, Asselain B, Castera L, De Pauw A (2009). Impact of BRCA1 and BRCA2 variants on splicing: clues from an allelic imbalance study. Eur J Hum Genet.

[23] Basic Local Alignment Search Tool. https://blast.ncbi.nlm.nih.gov/Blast.cgi. Accessed 12 Mar 2019.

[24] EMBOSS Water. https://www.ebi.ac.uk/Tools/psa/emboss_water. Accessed 12 Mar 2019.

[25] RepeatMasker. http://www.repeatmasker.org/. Accessed 12 Mar 2019.

[26] Lopez-Flores I, Garrido-Ramos MA (2012). The repetitive DNA content of eukaryotic genomes. Genome Dyn.

[27] Bacolla A, Tainer JA, Vasquez KM, Cooper DN (2016). Translocation and deletion breakpoints in cancer genomes are associated with potential non-B DNA-forming sequences. Nucleic Acids Res.

[28] RepeatAround. http://portugene.com/repeataround.html. Accessed 12 Mar 2019.

[29] QGRS MAPPER. http://bioinformatics.ramapo.edu/QGRS/analyze.php. Accessed 12 Mar 2019.

[30] FUZZNUC. http://emboss.bioinformatics.nl/cgi-bin/emboss/fuzznuc. Accessed 12 Mar 2019.

[31] Duan B, Wang X, Chu H, Hu Y, Huang X, Qu B (2003). Deficiency of factor XIII gene in Chinese: 3 novel mutations. Int J Hematol.

[32] Vysokovsky A, Saxena R, Landau M, Zivelin A, Eskaraev R, Rosenberg N (2004). Seven novel mutations in the factor XIII A-subunit gene causing hereditary factor XIII deficiency in 10 unrelated families. J Thromb Haemost.

[33] Wu S, Wang Z, Dong N, Bai X, Ruan C (2006). A novel compound heterozygous mutation in the F13A gene causing hereditary factor XIII deficiency in a Chinese family. J Thromb Haemost.

[34] Alavi SER, Jalalvand M, Assadollahi V, Tabibian S, Dorgalaleh A (2018). Intracranial hemorrhage: a devastating outcome of congenital bleeding disorders-prevalence, diagnosis, and management, with a special focus on congenital Factor XIII deficiency. Semin Thromb Hemost.

[35] Schenk VW (1963). Haemorrhages in spinal cord with Syringomyelia in a patient with Haemophilia. Acta Neuropathol.

[36] Wisoff JH, Rovit RL, Ho V, Leventhal H (1985). Spontaneous hematomyelia secondary to factor XI deficiency. Case report. J Neurosurg.

[37] Duckert F, Jung E, Shmerling DH (1960). A hitherto undescribed congenital haemorrhagic diathesis probably due to fibrin stabilizing factor deficiency. Thromb Diath Haemorrh.

[38] Wozniak G, Noll T (2002). Factor XIII and wound healing. Hamostaseologie.

[39] Soendergaard C, Kvist PH, Seidelin JB, Nielsen OH (2013). Tissue-regenerating functions of coagulation factor XIII. J Thromb Haemost.

[40] Inbal A, Lubetsky A, Krapp T, Castel D, Shaish A, Dickneitte G (2005). Impaired wound healing in factor XIII deficient mice. Thromb Haemost.

[41] Haroon ZA, Hettasch JM, Lai TS, Dewhirst MW, Greenberg CS (1999). Tissue transglutaminase is expressed, active, and directly involved in rat dermal wound healing and angiogenesis. FASEB J.

[42] Rodriguez-Merchan EC (2012). Surgical wound healing in bleeding disorders. Haemophilia.

[43] Schroeder V, Durrer D, Meili E, Schubiger G, Kohler HP (2007). Congenital factor XIII deficiency in Switzerland: from the worldwide first case in 1960 to its molecular characterisation in 2005. Swiss Med Wkly.

[44] Peyvandi F, Tagliabue L, Menegatti M, Karimi M, Komaromi I, Katona E (2004). Phenotype-genotype characterization of 10 families with severe a subunit factor XIII deficiency. Hum Mutat.

[45] Biswas A, Ivaskevicius V, Thomas A, Varvenne M, Brand B, Rott H (2014). Eight novel F13A1 gene missense mutations in patients with mild FXIII deficiency: in silico analysis suggests changes in FXIII-A subunit structure/function. Ann Hematol.

[46] Izumi T, Nagaoka U, Saito T, Takamatsu J, Saito H, Ichinose A (1998). Novel deletion and insertion mutations cause splicing defects, leading to severe reduction in mRNA levels of the A subunit in severe factor XIII deficiency. Thromb Haemost.

[47] Zhang F, Khajavi M, Connolly AM, Towne CF, Batish SD, Lupski JR (2009). The DNA replication FoSTeS/MMBIR mechanism can generate genomic, genic and exonic complex rearrangements in humans. Nat Genet.

