# Supplementary material for: Phenotype and genotype of FXIII deficiency in two unrelated probands: identification of a novel F13A1 large deletion mediated by complex rearrangement
Source: Orphanet J Rare Dis. 2019 Jul 24;14:182. doi: 10.1186/s13023-019-1144-z (PMC6657060; doi:10.1186/s13023-019-1144-z)
Supplement: Supplementary file 1 — Table S1. The target sequences for CNV detection of F13A1 and F13B in probands 1 and 2. Table S2. Primer sequences for quantitative primer walking detection and LR-PCR amplification. Table S3. Quantitative PCR results of 10 fragments in intron 6 and intron 8 of F13A1 for the primer walking detection in proband 2. Table S4. Primer sequences for mRNA analysis of the large deletion of F13A1. Figure S1. Effect of the F13A1 large deletion on transcription analyzed by RT-PCR in proband 2. Figure S2. Instability analysis of the DNA sequences around breakpoints and insertion in proband 2. (DOCX 4664 kb) [file 13023_2019_1144_MOESM1_ESM.docx]

**Additional file 1: Table S1. The target sequences for CNV detection of *F13A1* and *F13B* in probands 1 and 2.**

| **Primers** | **Targets** | **Target locations** | **Target sequences** |
| --- | --- | --- | --- |
|  |  |  |  |
| *F13A1*: GRCh37.p13.Chr6 | | | |
| A-5’-1 | 5’ UTR | 6330494-6330551 | TGACAGACTTTGTGATGAGTAGGAAATTGTATCTATCTTTAAGGCCTGCAGTCAAGAA |
| A-5’-2 | 5’ UTR | 6327305-6327360 | CCACCCATCCCAAGTATCTCACTTTGCCAGAGGAAAAATTCAATGATAACAAAACA |
| A-5’-3 | 5’ UTR | 6325379-6325420 | GACGTGAACTCCCTCTGGCTGCACGGGCCTGGGCATACCTTC |
| A-5’-4 | 5’ UTR | 6323976-6324031 | TTTACAGGGTATTCCTCATGGTTATATGGCTGAGACATCAGAACAAAGGTTTCTGG |
| A-5’-5 | 5’ UTR | 6321377-6321420 | TGCAAATAAGGCATGGTCTGCATTCCTGAATCATCCCAGAGCCA |
| A-E1-1 | E 1 | 6320912-6320961 | GACAGGGGAGGAGGGTGAGGGCTCCTCTTAGGAAGTTATTTAAGAGCCAA |
| A-E1-2 | E 1 | 6320869-6320910 | GCCTCGGGGACTTCCTCAAACGGACTCGGGAAAGACAAGACA |
| A-E2-1 | E 2 | 6318841-6318886 | TTCCAGGACCGCCTTTGGAGGCAGAAGAGCAGTTCCACCCAATAAC |
| A-E2-2 | E 2 | 6318796-6318835 | GCCCTGAAGCTCCACTGTGGGCAGGTCATCTTCCGCTGCA |
| A-E3-1 | E 3 | 6305657-6305707 | CACACTGACAAGTATGAAAACAACAAGCTGATTGTCCGCAGAGGGCAGTCT |
| A-E3-2 | E 3 | 6305604-6305655 | AAGAGATCCCTTCTGGGGTCATATGGACGACTGAAGTCAATCTGCACATAGA |
| A-E4-1 | E 4 | 6266967-6267020 | GGGAACCTACATCCCAGTGCCTATAGTCTCAGAGTTACAAAGTGGAAAGTGGGG |
| A-E4-2 | E 4 | 6266846-6266893 | TCGAAGTACGCCATAGGGAGTCCAGACAGCAACATACATGCGGAATTT |
| A-E5-1 | E 5 | 6251121-6251173 | CCCTGATGCAGATGATGCTGTGTATCTGGACAATGAGAAAGAAAGAGAAGAGT |
| A-E5-2 | E 5 | 6251018-6251071 | ACAATAACAAATTTTAAGTGGCTCACCTGACCATAGCTCCAGCTTCTGGTCTTG |
| A-E6-1 | E 6 | 6248597-6248642 | GCATCCTGGACACTTGCCTGTATGTGATGGACAGAGCACAAATGGA |
| A-E6-2 | E 6 | 6248550-6248594 | CAGACCCCACACGGCTGACTTTGATGGGATTCCCTCTTCCAGAGA |
| A-E7-1 | E 7 | 6225066-6225115 | CACTTCTCACGGACTCATTTAGGTGAATGCCAAAGATGACGAAGGTGTCC |
| A-E7-2 | E 7 | 6224908-6224959 | TGGATACTTACATGTGTTAAAGACACCAGCAAAAACCCAGCATTGGCCATAC |
| A-E8-1 | E 8 | 6222384-6222439 | TGATGTGTTTAGCTGTGGTCTGTCCTTTCCTGTAGTTTTACGATGCCTTGGAATAC |
| A-E8-2 | E 8 | 6222267-6222316 | ACACTGAATCCTTGGTGAGTTTGGAATTCACGTTCCCATCTTCTTCCAGG |
| A-E9-1 | E 9 | 6197536-6197589 | TTTAACCTTTCTGGGCTTGTGTTTTCTAAGGAACTACCACTGCTGGAATGAAGC |
| A-E9-2 | E 9 | 6197489-6197530 | CTTGCCAGCCTCCAAATCCAACAGGAAGGTCAGGCCTTGTCA |
| A-E10-1 | E 10 | 6196099-6196139 | CCCTTCCTCTGTGCAATGCAGGCATGTATCGGTGTGGCCCC |
| A-E10-2 | E 10 | 6196048-6196091 | TGCATCAAATTGGAAGCAGACATGGCCGTGCTTGATGGCTTGAA |
| A-E11-1 | E 11 | 6182324-6182377 | CAGGTCAACAGCGACCTCATTTACATTACAGCTAAGAAAGATGGCACTCATGTG |
| A-E11-2 | E 11 | 6182265-6182314 | CCTCCAATTTGTTTGGTCACAATTAATTTCCCAATGTGGGTGGCATCCAC |
| A-E12-1 | E 12 | 6175087-6175142 | GGATTGTATTTTTGCCTGTCATTATCTCTGGATCTCCCCCAGGTCAAGAAGAAGAG |
| A-E12-2 | E 12 | 6175030-6175079 | TTCTGTGTTGAGGGGCTTTTTAGCTCCGTACATCAGGGCAGTTTCTAGGG |
| A-E13-1 | E 13 | 6167769-6167812 | TGGGTCAGCTGCTGGAACAAGCGTCCCTGCACTTCTTTGTCACA |
| A-E13-2 | E 13 | 6167708-6167751 | GGATGGTTAGCACGGTGGACTTTTGCTTGGCCAGAACATCCCTG |
| A-E14-1 | E 14 | 6152143-6152194 | TCTCTCTCTTAGGTCCGTGGCACTCAGGTAGTTGGTTCTGACATGACTGTGA |
| A-E14-2 | E 14 | 6152035-6152086 | CCAAGGTTTACCGGAACATCTTCTTCATTGGTCTTGTTACTCCAGGACCATC |
| A-E15-1 | E 15 | 6145934-6145974 | GAAGAAGTGTGCCGGCCCTGGGTCTCTGGGCATCGGAAGCT |
| A-E15-2 | E 15 | 6145885-6145928 | CAGCTCGCCATACACATGTCTCAGGGAGTCACTGCTCATGCTGG |
| A-3’-1 | 3’ UTR | 6145482-6145535 | GGCTCTCTTACCAGGGAGATTTGCTCAATACCTGGCCTCATTTAAAACAAGACT |
| A-3’-2 | 3’ UTR | 6144630-6144688 | GAATCACAGTCTACTGACCTAAATCACACCCTAGACATATCAGAGGGAAATTCTGACCA |
| A-3’-3 | 3’ UTR | 6144368-6144433 | CCAATATCCAATTTTAAAATGAAATGCATTTTGCTAGACAGTTAAACTGGCTTAACTTAGTATATT |
| A-3’-4 | 3’ UTR | 6144233-6144298 | GAGGAAGGATGGTTTCTCAAGAAGTTGTGATTAGTAGTGTGTTATATTGAAAATTAAAAGCATTCA |
| *F13B*: GRCh37.p13.Chr1 | | | |
| B-5’-1 | 5’ UTR | 197036777-197036848 | TGAAATATGTTGTTGCCTAGACTGCACTCCTTAAAGAGATACAGAACCATAGTTGTCTCAATGATAGAATAG |
| B-1-1 | E 1 | 197036345-197036406 | CAAGTTCCTAGTGCTTAGAATTGTTAAAATCTTTGTGAAGCACACCACTGAAGATGAGGTTG |
| B-1-2 | E 1 | 197036274-197036343 | TAAGAATTAATTTTACCTTCTGCATAGAGTTCTCCTGAGATTATCAATATGATGATAAAAGTCAGGTTTT |
| B-2-1 | E 2 | 197032133-197032186 | GAAACCCTGTGGTTTTCCTCATGTGGAAAATGGAAGAATTGCCCAATATTACTA |
| B-2-2 | E 2 | 197032010-197032061 | CAGCCTTCTGTTGTACACGTGGTTTGCTCTTCTTGTCTTCCACTTTCAGTGG |
| B-3-1 | E 3 | 197031054-197031109 | TTTCCCATAGAAAAATGCACTAAGCCTGACCTGAGTAATGGTTACATCTCTGATGT |
| B-3-2 | E 3 | 197030967-197031012 | ACCACTTCTTCATCCTTCCCTCCAGTGGTTTTGTACCCTGAAGCGC |
| B-4-1 | E 4 | 197030092-197030147 | CAAAGTGAAGGACAAAGTACAATACGAATGTGCTACTGGCTACTACACAGCTGGAG |
| B-4-2 | E 4 | 197030010-197030063 | ATTCCAAATGAGAACCTACTGGTACATTTTGGTGTGAGAGACCATCCGTATGTG |
| B-5-1 | E 5 | 197029565-197029623 | TGTAAAGCAAACCTATGAAGAAGGAGATGTCGTTCAGTTTTTCTGTCATGAAAATTATT |
| B-5-2 | E 5 | 197029452-197029511 | GATAAAGACATGGCATTTTGCGAGTATTAAATTTAAAAATTTACCTTCGCATACAGGAGA |
| B-6-1 | E 6 | 197026505-197026563 | TGAACTATTTCTCCATGACGATAAGTTGTTGAATGTGTTTGAATTTTGGAGTTTATGGG |
| B-7-1 | E 7 | 197026333-197026391 | AAAGGTACAGTTGAAAGAGAACTGACCATTTAGCTACACATGCAGATTTCATTTTAGCA |
| B-7-2 | E 7 | 197026154-197026205 | GGAGGAAGTGTCCATTTTCCACGATTACAAGTTATCTCATTCGATCCATGGA |
| B-8-1 | E 8 | 197024917-197024966 | ATTGGCAAGCTATGCAACAGGATCCTCAGTGGAATATAGATGCAATGAAT |
| B-8-2 | E 8 | 197024860-197024907 | GGGATGACCATTTTCCTTGTTCGCAACGAGATATTTTTGATCCCCTCA |
| B-9-1 | E 9 | 197021846-197021910 | GGAAATATGAAGGGAAAGTCTTACATGGAGATTTAATAGATTTTGTATGTAAACAGGGATATGAC |
| B-9-2 | E 9 | 197021783-197021840 | ATATTTCACTTCTCCTCTGTTGCACTGCACAGATAATTCAGACAATGGGGTTAATGGA |
| B-10-1 | E 10 | 197019988-197020059 | GGATGTTCATTATAGCAATTCATTGTATACTTTAAAACTTATTTTTGCAGAATCTAAAGGAATGTGCACATC |
| B-10-2 | E 10 | 197019860-197019923 | CTAAACAATAGGCCTCCCTAGATCCTTCTAGGAAATGGTGATCAAAACATCTGTATTCTACTGA |
| B-11-1 | E 11 | 197009799-197009862 | CATGCACATTATCTTTTACTGAAATGGAAAAGAATAATTTACTTCTGAAATGGGATTTTGACAA |
| B-11-2 | E 11 | 197009717-197009784 | TCCAGTAATATATAATTCAGCTGGATAAGTATCTCCTCTACAAATAAACTCAATATATTCACCATGCA |
| B-12-1 | E 12 | 197008536-197008593 | CACCTGACTGCAATTGATGCTTATTTCAAAAATCTCTCTTTTTCCCCTCAAGCACTCT |
| B-12-2 | E 12 | 197008437-197008508 | TCCTCAAAATTATATTTTATAAGGAATTTCATGATGTATTGAAATATGACTCCTCTTTCTGCCATTCATTTC |

E, exon; 5’ UTR, 5’ untranslated region; 3’ UTR, 3’ untranslated region; CNV, copy number variation.

**Table S2. Primer sequences for quantitative primer walking detection and LR-PCR amplification.**

| **Fragments** | **F/R** | **Sequences (5’-3’)** | **Targets** | **Product sizes (bp)** |
| --- | --- | --- | --- | --- |
| Quantitative primer walking detection of *F13A1* | | | | |
| I6-1 | F | GCACAGAAGATGTGCAAGGC | g.77737- g.77960 | 224 |
|  | R | GCTGTCCTTACCCTGTGTCC |  |  |
| I6-2 | F | CTGTTTCGCCCACAAGATTTCTC | g.78740-g.79025 | 286 |
|  | R | TAACAGCTTCCGCTTAGCCAG |  |  |
| I6-3 | F | CTTGGAAGTGGTGTCCCTCA | g.81890-g.82292 | 403 |
|  | R | TGGTACCTGATCGTCTTTGGAA |  |  |
| I6-4 | F | TCTGTGCTAGCCCTGGGAAT | g.87874-g.88330 | 457 |
|  | R | TGCCTGGCTCTTCATCCTTG |  |  |
| I6-5 | F | GGGGATACGCTCAGCTTGTATTA | g.96803-g.97364 | 562 |
|  | R | TGGAGTAAAGCAGAGAGGCAAC |  |  |
| I8-1 | F | TTAGCTGCAGTCCAAACAGC | g.107312-g.107811 | 500 |
|  | R | TAGATTAAGACCACCCATTGCAG |  |  |
| I8-2 | F | ATCACCTGGAGTTAGACC | g.114336-g.114516 | 198 |
|  | R | CATGCTGTTAAGAGGTAGA |  |  |
| I8-3 | F | CCAACTCTGGCAACCTC | g.115852-g.115998 | 146 |
|  | R | ACAACACGGACGAACCT |  |  |
| I8-4 | F | GCCCCTTCAGGCATTAT | g.118251-g.118537 | 287 |
|  | R | TCTCACCCCACCTCCAC |  |  |
| I8-5 | F | GCCAGAGTGGTAAAAGGGGG | g.119713-g.120283 | 571 |
|  | R | TGCAGTTCTACCAGGGCATC |  |  |
| R1 | F | AGAGGATGCCAAGTGCTGAC |  | 460 |
| (*POLR2A*) | R | TGAGAGCCAGAGATCCACGA |  |  |
| R2 | F | CCACAACACTTCTCTGCCCT |  | 481 |
| (*TBX15*) | R | TGGGGCTAGCCAGCTCTAAA |  |  |
| R3 | F | TTTACAAAAACCCTTCCGAG |  | 215 |
| (*RPP14*) | R | TCCTTCACAGCCGAAATAA |  |  |
| LR-PCR amplification | | | | |
|  | F | CCATCAAAGTCAGCCGTGTG | g.77349-g.118494 | ~ 2kb |
|  | R | GTAGCTTTGCAAACTTGCAGCC |  |  |

F/R, forward primer/reverse primer; I6, intron 6; I8, intron 8; R1, R2 and R3, three fragments located on the reference genes of *POLR2A*, *TBX15*, and *RPP14*, respectively.

**Table S3. Quantitative PCR results of 10 fragments in intron 6 and intron 8 of *F13A1* for the primer walking detection in proband 2.**

| **Primer pairs** | **Samples’ mean Ct values** | |  | **Absolute CNVs** | | | **Mean CNVs** |
| --- | --- | --- | --- | --- | --- | --- | --- |
|  | **N** | **P2** |  | **R1** | **R2** | **R3** |  |
| I6-1 | 22.77 | 21.90 |  | 0.88 | 0.96 | 0.93 | 0.92 |
| I6-2 | 24.08 | 23.40 |  | 1.00 | 1.10 | 1.06 | 1.06 |
| I6-3 | 22.60 | 22.00 |  | 1.06 | 1.16 | 1.13 | 1.12 |
| I6-4 | 22.26 | 21.61 |  | 1.03 | 1.13 | 1.09 | 1.08 |
| I6-5 | 22.22 | 21.41 |  | 0.92 | 1.00 | 0.97 | 0.97 |
| R1 | 23.65 | 23.96 |  |  |  |  |  |
| R2 | 21.01 | 21.19 |  |  |  |  |  |
| R3 | 24.35 | 24.58 |  |  |  |  |  |
| I8-1 | 21.70 | 20.71 |  | 0.96 | 1.05 | 1.04 | 1.02 |
| I8-2 | 22.04 | 20.89 |  | 0.86 | 0.94 | 0.94 | 0.91 |
| I8-3 | 23.56 | 22.64 |  | 1.01 | 1.11 | 1.10 | 1.07 |
| I8-4 | 20.64 | 20.68 |  | 1.96 | 2.15 | 2.14 | 2.09 |
| I8-5 | 22.68 | 22.73 |  | 1.97 | 2.16 | 2.15 | 2.09 |
| R1 | 20.94 | 21.01 |  |  |  |  |  |
| R2 | 19.54 | 19.48 |  |  |  |  |  |
| R3 | 22.62 | 22.57 |  |  |  |  |  |

I6, intron; I8, intron 8; N, normal control; P2, proband 2; Absolute CNVs= 2 x Relative CNVs (patient)/Relative CNVs (standard), Relative CNVs=2^(-∆Ct values), ∆Ct values=Ct(target regions)-Ct(reference).

**Table S4. Primer sequences for mRNA analysis of the large deletion of *F13A1*.**

| **Fragments** | **F/R** | **Sequences (5’-3’)** | **Targets** | **Product sizes (bp)** |
| --- | --- | --- | --- | --- |
| mRNA transcript analysis | | | | |
| E4-E11 | F | GTCATTGGTCGCTACCCACA | c.313-c.1474 | 1162 |
|  | R | TCTCTTCTTCTTGACCTTCTTGGA |  |  |
| SNP flanking sequence amplification | | | | |
|  | F | CTCCATCACCTTCCGGAACAAC | c.1608-c.1728 | 121 |
|  | R | CACGTCGAACGTCTCCTTCTTG |  |  |
| SNaPshot single nucleotide extension | | | | |
|  | EP | TTTTCACCTTCTACACCGGGGTCC | c.1674-c.1693 | 25 |

F/R, forward primer/reverse primer; E4-E11, exon 4 through exon 11; EP, extension primer.

**
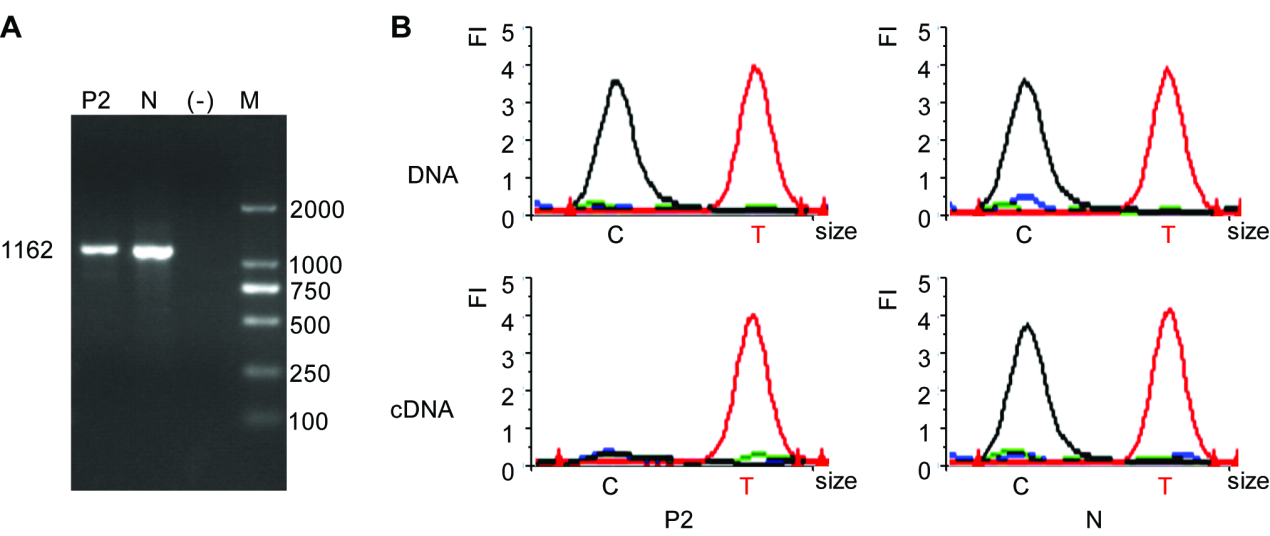
**

**Figure S1. Effect of the *F13A1* large deletion on transcription analyzed by RT-PCR in proband 2.** A, Electrophoresis of the transcripts obtained by RT-PCR amplification in proband 2 and normal control; B, Results of allelic imbalance detection with DNA and cDNA templates in proband 2 and normal control, both were heterozygotes of the SNP (c.1694C>T; rs5982). FI, fluorescence intensity; P2, proband 2; N, normal control; (-) negative control; M, marker; C, SNP-C; T, SNP-T.


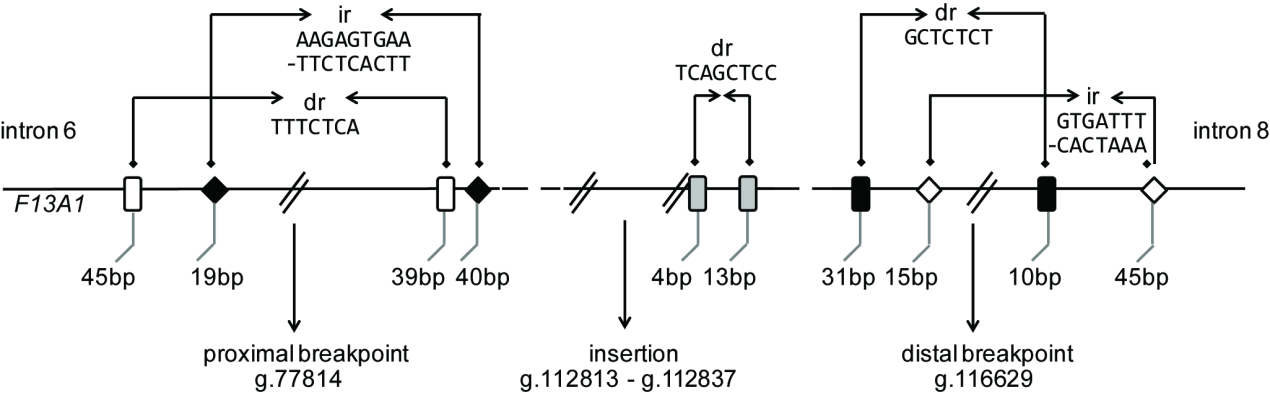


**Figure S2. Instability analysis of the DNA sequences around breakpoints and insertion in proband 2.** Breakpoints indicated by double slashes; Abbreviated long sequences between intron 6 and intron 8 of F13A1 indicated by dotted lines; Distances to the nearest breakpoint is indicated under the line; dr, direct repeat; ir, inverted repeat.
